# “Chiclero’s Ulcer” Due to *Leishmania mexicana* in Travelers Returning from Central America: A Case Report and Review of the Literature

**DOI:** 10.3390/pathogens10091112

**Published:** 2021-08-31

**Authors:** Carole Eldin, Coralie l’Ollivier, Stephane Ranque, Philippe Gautret, Philippe Parola

**Affiliations:** 1 Aix Marseille Université, Institut de Recherche pour le Développement, Assistance Publique-Hôpitaux de Marseille, Service de Santé des Armées, VITROME: Vecteurs-Infections Tropicales et Méditerranéennes, 13385 Marseille, France; carole.eldin@ap-hm.fr (C.E.); stephane.ranque@ap-hm.fr (S.R.); philippe.gautret@ap-hm.fr (P.G.); philippe.parola@ap-hm.fr (P.P.); 2 IHU Méditerranée Infection, 13385 Marseille, France

**Keywords:** *Leishmania mexicana*, Chicleros, diagnosis, treatment

## Abstract

Cutaneous leishmaniasis (CL) due to a New World species of Leishmania is increasingly seen among returning international travelers, and most cases arise from travel to Mexico, Central and South America. We described a case of CL in a women presenting a nonhealing ulceration under her right ear with slight increase of size of the left parotid gland under the skin lesion, evolving for 4 months. In her history of travel, she reported a ten-day stay in Mexico during the Christmas vacation in the Yucatan region with only half a day walking in the tropical forest. Diagnosis of CL due to *Leishmania mexicana* was done via PCR detection and sequencing from swab sampling of the lesion. The patient recovered without antiparasitic treatment. Clinicians should consider diagnosing Chiclero’s ulcer in patients returning from endemic areas such as Central America and Texas who present with chronic ulceration. A noninvasive sampling is sufficient for the PCR-based diagnosis of this disease.

## 1. Introduction

Cutaneous leishmaniasis (CL) due to *Leishmania* species of the New World is increasingly seen among returning international travelers, and most cases arise from travel to Mexico, Central and/or South America [1]. In Mexico and Central America, *Leishmania mexicana* is endemic and extends north into central Texas [2]. The vectors of this infection are Lutzomyia sand flies that transmit *Leishmania* among different mammalian reservoirs (rodents, opossums, armadillos, cats and dogs) [2]. The typical clinical presentation of *L. mexicana* infection is called the “Chiclero’s ulcer”, because it was first described in Mexican “Chicleros”: forest workers of the Yucatan peninsula who collected the gum of chicozapote tropical trees that was used in the confection of chewing gum (“Chiclets” in Spanish) [3]. The clinical presentation is characterized by a single ulceration classically associated with involvement of the ear [3]. We report a case of Chiclero’s ulcer in a traveler returning from Mexico. A review of the literature about CL due to this species in travelers was conducted.

## 2. Case Report and Review of the Literature

**Figure 1:**
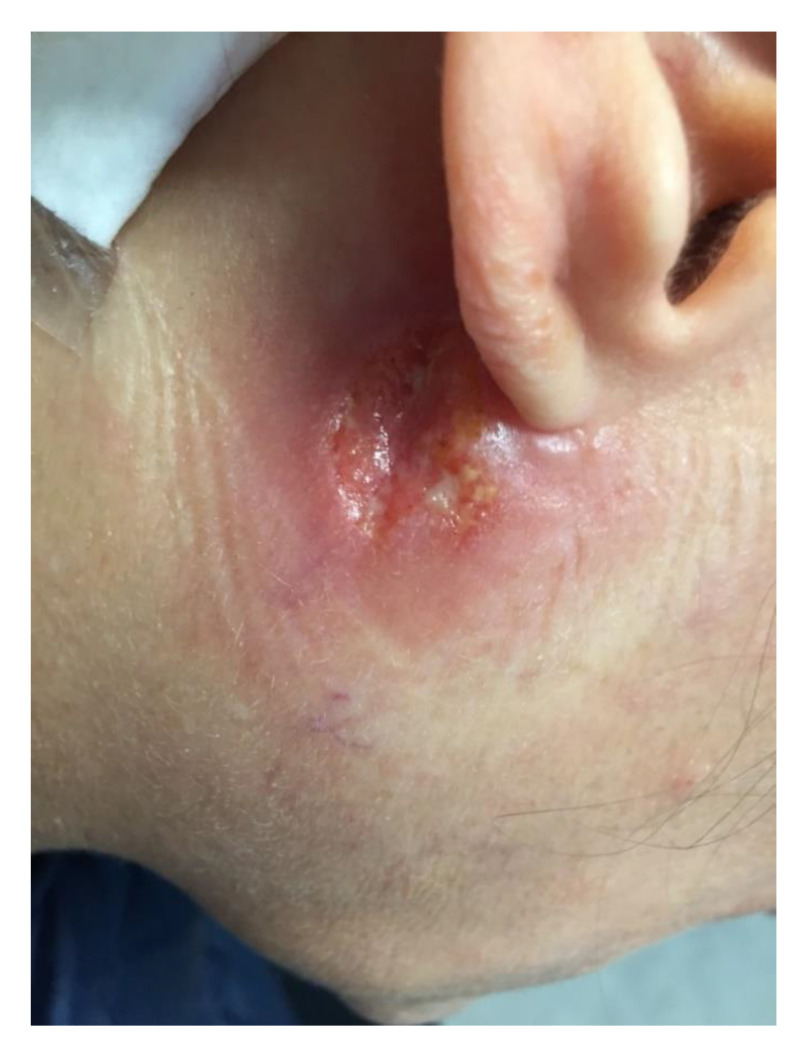
Nonhealing ulcer due to *Leishmania mexicana*, localized under the right ear in a patient 4 months after returning from a travel to Mexico.

A previously healthy 44-year-old woman presented to the infectious diseases outpatient department of the University Hospital (IHU Méditerranée Infection) of Marseille, France, in May 2019 for a non-healing ulcer located under her right ear slightly extending to the lower part of the ear lobe, which evolved since the end of January (Figure 1). 

**Figure 2:**
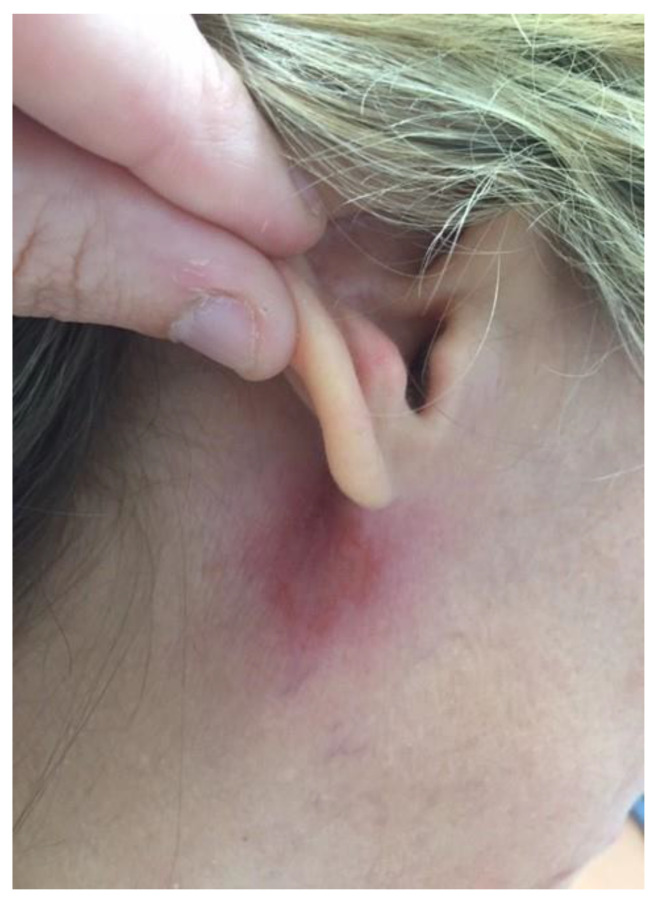
Evolution of the skin lesion 1 month later without treatment.

Regarding her travel history, she reported a stay of ten days in Mexico during the Christmas vacation in the Yucatan region with only half a day walking in the tropical forest. She reported paresthesia in the area surrounding the lesion. With a the cervical and thoracic CT-scan, there was no lymphadenitis observed but a slight increase of size of the left parotid gland under the skin lesion. Routine laboratory tests were normal, including C-reactive protein level and blood cell counts. Swab samples of the ulcer were collected for microbiological investigations. Polymerase chain reaction (PCR) and culture aimed at detecting Mycobacterium spp, dermatophytes and pyogenic bacteria were negative. Real-time PCR tests targeting the kinetoplastic minicircle gene successfully detected *Leishmania* spp. [4]. Subsequent sequencing targeting both the ITS1 and ITS2 region of the rRNA gene performed by the laboratory of the National Reference Center—Laboratory Expert (CNRL) for *Leishmania* (Montpellier, France) found 100% identity with *L. mexicana* (Genbank FJ948435.1). Serological investigation showed a positive serology for *Leishmania* spp. only on immunoblotting assay (LD-Bio Diagnostic, Lyons, France). The diagnosis of cutaneous leishmaniasis due to *L. mexicana* was retained. At a one month of follow-up, the patient had recovered and skin ulceration and inflammation had disappeared (Figure 2).

We performed a review of the literature in Medline in the English language using the following keywords “Cutaneous leishmaniasis”, “*Leishmania mexicana*” AND “travel” OR “traveler”. We found 12 references reporting a total of twenty-eight humans cases of CL caused by *L. mexicana* in travelers (Table 1) [1,3,5,6,7,8,9,10,11,12,13]. Most cases were reported in male travelers returning from Belize, followed by Mexico. Two references reported cases in soldiers who underwent jungle military training in Belize [1,8]. The others reported travels were mainly for touristic reasons. Except for the present case, none involved a French traveler. Regarding clinical features, only three patients presented with the typical localization involving the ear, and three patients had lesions on the face (nose and eyelid). For all patients except our case, the diagnosis was performed on a skin biopsy. The reported treatment strategies were highly heterogeneous. Only two cases, (including ours) received no treatment and healed spontaneously. The other treatments reported in the literature included liposomal amphotericin B, intravenous and intralesional sodium stibogluconate, IV or oral fluconazole, cryotherapy, thermotherapy and topical imiquimod. Two patients had recurrence of their lesions after a first line of treatment and required a second-line treatment for healing.

## 3. Discussion

We detected and identified *L. mexicana* in a French traveler returning from Mexico by using PCR-based methods on a swab sample from the ulcer. All previous cases had been diagnosed on a skin biopsy sample. Swabbing of ulcers or eschars is a noninvasive, highly sensitive, diagnostic method that advantageously avoids performing a skin biopsy [14]. It is noteworthy that antibody detection by immunoblotting detected antibodies directed against the 14 and 16 kDa *L. infantum* antigens, which are present in the *Leishmania* species of the Vianna complex [15]. In a recent report of the Geosentinel network, *L. mexicana* was found in 3.6% of 274 returned travelers presenting with CL, confirming that it is a relatively rare but emerging CL species in travelers [1]. For clinicians, the main issue about CL in travelers returning from Central and South America is to assess the risk of mucocutaneous or mucous leishmaniasis, which are dilapidating and difficult to treat diseases. In the reported cases of CL due to *L. mexicana*, involvement of the ear or nose may have prompted clinicians to treat it aggressively because of a putative risk of mucocutaneous involvement. However, analyzing the literature on *L. mexicana* CL is reassuring because neither lymphatic nor mucosal involvement has been reported with this species [3]. The majority of cases heal spontaneously after 3–9 months [3]. Spontaneous healing occurred after 5 months in our patient, and after 11 months in the other untreated case reported in the literature [3]. However, recurrences were observed in two patients after systemic intravenous treatment (liposomal amphotericin B and IV sodium stilbogluconate) [7,10]. IV or oral fluconazole was not recommended, whereas it was administrated in two cases. The efficiency is difficult to evaluate because the therapeutic scheme included two other successive treatments with different molecules [16]. In fact, recent guidelines about the treatment of CL due to *L. mexicana* in travelers recommend abstention in patients presenting with up to three lesions if there is no aesthetic disfigurement risk [16]. In patients presenting with more than three lesions, local therapy (topic or intralesional) is recommended [16]. In case of recurrence, lesions with sensitive localization, and/or a larger than 30 mm diameter, the first-line systemic treatment recommended is ketoconazole [16]. Yet, we should stress that ketoconazole has been administered to only one of the 28 reported cases. Protection against sandfly bites, including avoidance of outdoor activities from dusk to dawn, appropriate clothes, use of indoor fans, use of DEET (N,N-diethyl-meta-toluamide) repellents or pyrethroid impregnated bednets, remain the only ways to prevent leishmaniosis in endemic countries.

## 4. Conclusions

Clinicians should consider Chiclero’s ulcer in patients presenting with a chronic ulceration and returning from endemic areas, including Central America and Texas. A noninvasive swab sampling of the ulceration is enough for the PCR-based microbiological diagnosis of CL due to *L. mexicana*.

## Figures and Tables

**Table 1:** Cases reports of *Leishmania mexicana* in travelers, review of literature.

Number of Cases	Ref	Sex, Age	Country of Travel	Country of Residence	Reason for Travel	Estimation of Incubation Time (Weeks)	CL Localisation	Sampling for Diagnosis	Treatment	Final Follow-Up
1		W, 44	Mexico, Yucatan	France	tourism	12	under the ear	swab	no	recovered after five months
10	[1]	ND	Belize (4), Mexico (4), Honduras (1)	ND	ND	ND	ND	ND	ND	ND
1	[3]	M, 86	Guatemala	USA	tourism	12	ear	biopsy	no	recovered after 11 months
1	[5]	M, 19	Belize	UK	military training	6	neck	biopsy	appropriate medical treatment	recovered
8	[6]	ND	Belize	The Netherlands	military training	ND	neck	biopsy	IV or IL Sb; One patient with unsatisfactory response, further treatment with lipid associated amphotericin B	recovered after 5 to 9 months
1	[7]	M, 42	Belize	Canada	university field school	11	ear, pinna	biopsy	liposomal amphotericin B then oral fluconazole, then IL Sb	recovered after five months
1	[8]	M, 23	Costa Rica	ND	tourism	8	leg, arm	biopsy	liposomal amphotericin B	recovered
1	[9]	ND	Bolivia and Peru	Israel	tourism	ND	nose	biopsy	IV or IL Sb	recovered
1	[10]	M, 50	Belize	USA	tourism	ND	eyelid	biopsy	IV Sb	recurrence, new treatment, recovered after 7 weeks
1	[11]	M, 64	Mexico, Yucatan	USA	tourism	ND	arm	biopsy	cryotherapy, thermotherapy , imiquimod 5% cream	recovered after several weeks,
1	[12]	M, 22	Oaxaca, Mexico	ND	tourism	20	nose, ear	biopsy	imiquimod 5% cream	recovered after 12 weeks
1	[13]	M, 39	Yucatán, Quintana Roo, and Baja California in Mexico	Taïwan	tourism	16	eyebrown	biopsy	IV fluconazole then liposomal amphotericin B then oral ketoconazole	recovered after 18 weeks

UK: United Kingdom; USA: United States of America; ND: not done; IL: intralesional; Sb: sodium stilbogluconate; IV: intraveinous; CL: cutaneous leishmaniasis; M: man; W: woman.

## Data Availability

All data are available within the article.
